# Lactic acidosis incidence with metformin in patients with type 2 diabetes and chronic kidney disease: A retrospective nested case‐control study

**DOI:** 10.1002/edm2.170

**Published:** 2020-07-17

**Authors:** Carlos A. Alvarez, Ethan A. Halm, Mary Jo V. Pugh, Darren K. McGuire, Sean Hennessy, Richard T. Miller, Ildiko Lingvay, Scott M. Vouri, Andrew R. Zullo, Hui Yang, Matt Chansard, Eric M. Mortensen

**Affiliations:** ^1^ Texas Tech University Health Sciences Center School of Pharmacy Dallas TX USA; ^2^ University of Texas Southwestern Medical Center Dallas TX USA; ^3^ Veterans Affairs North Texas Health Care System Dallas TX USA; ^4^ University of Utah School of Medicine Salt Lake City UT USA; ^5^ University of Pennsylvania Perelman School of Medicine Philadelphia PA USA; ^6^ University of Florida College of Pharmacy Gainesville FL USA; ^7^ Brown University School of Public Health and Providence Veterans Affairs Medical Center Providence RI USA; ^8^ University of Connecticut School of Medicine Farmington CT USA

**Keywords:** chronic kidney disease, diabetes mellitus, lactic acidosis, metformin

## Abstract

**Objective:**

Compare rates of lactic acidosis (LA) among metformin‐exposed and unexposed patients with type 2 diabetes mellitus and varying degrees of chronic kidney disease (CKD).

**Research Design and Methods:**

Retrospective, nested case‐control study using data from national VA Corporate Data Warehouse. All adult patients with type 2 diabetes and CKD newly dispensed any antihyperglycaemic medication during FY 2003‐13 were included. The outcome was LA hospitalization or serum lactate >5 mEq/L. Exposure to metformin was evaluated in the three months prior to event. Estimates were adjusted for 31 covariates, including demographics, comorbidities and medications.

**Results:**

Overall, 320 882 patients were included, contributing a total of 1 331 784 person‐years of follow‐up. LA occurred in 2 665 patients, generating an overall incidence rate of 2.00 (95% CI 1.93‐2.08) per 1000 person‐years. Metformin exposure in the prior 3 months was associated with an elevated adjusted hazard of LA (HR 1.97, 95% CI 1.69‐2.29). No association was evident in patients with CKD stage 1 or 2 (HR 1.05, 95% CI 0.71‐1.57), but associations were present and progressively greater in patients with CKD stage 3a through 5: HR 3.09, 95% CI 2.19‐4.35 in CKD 3a, HR 3.34, 95% CI 1.95‐5.72 in CKD 3b, HR 7.87, 95% CI 3.51‐17.61 in CKD stage 4&5.

**Conclusion:**

Metformin was not associated with an elevated risk of LA in persons with stage 1‐2 CKD, but was associated with a progressively higher risk at more advanced stages of CKD.

## Abstract 2

Metformin was not associated with an elevated risk of LA in persons with stage 1‐2 CKD, but was associated with a progressively higher risk at more advanced stages of CKD.

## 1 INTRODUCTION

Metformin is the most prescribed medication for patients with type 2 diabetes mellitus (T2DM) and considered first line therapy by international professional society recommendations.^1^, ^2^, ^3^ Metformin lowers blood glucose, is associated with a low risk of serious hypoglycaemia and does not promote weight gain.^4^ It lowered cardiovascular risk in overweight or obese patients with T2DM in one trial,^5^ though some uncertainty persists about its net cardiovascular benefit.^6^ Until 2016, the Food and Drug Administration (FDA) labels for metformin contained a contraindication for men with serum creatinine of ≥1.5 mg/dL and women with serum creatinine ≥1.4 mg/dL and a boxed warning about the risk of lactic acidosis (LA) associated with renal impairment.^7^ The contraindication and warning were implemented due to increased LA risk observed in patients receiving phenformin, the predecessor to metformin in the biguanide class that was removed from the US market in 1977.^8^ Since metformin is eliminated unchanged by the kidneys, it was feared that elevated metformin concentrations in patients with CKD might also increase the risk for LA.^9^

Despite these concerns, some clinicians have been prescribing metformin to persons with renal insufficiency because perceived benefits outweigh the risks compared with other antihyperglycaemic medications.^10^ Estimates from observational data suggest that 30% of patients receiving metformin had product‐labelled contraindications.^11^, ^12^ In April 2016, largely in response to two Citizen Petition to the FDA and supported by a systematic review evaluating associations between metformin and kidney function,^13^ the FDA relaxed the renal restrictions for metformin, switching from a serum creatinine‐based contraindication to one that uses estimated glomerular filtration rate (eGFR). The change permits metformin initiation in individuals with an eGFR of 45 mL/min/1.73 m^2^ (Stage 3a) or higher and continued use with closer monitoring down to eGFR 30 mL/min/1.73 m^2^ (Stage 3b). As a result of this change, an estimated one million additional US patients with type 2 diabetes are eligible to receive metformin.^13^ These Citizen Petitions were based on a series of systematic reviews of trials that demonstrated a low incidence of LA in persons with type‐2 diabetes that was not associated with metformin use^14^, ^15^, ^16^, ^17^ and supported by a systematic review published subsequently focusing specifically on patients with kidney disease.^18^ Metformin's potential association with LA was further evaluated in the real‐world setting in an observational cohort studies across a range of eGFR.^19^ Metformin was found to be associated with all cause acidosis in patients with an eGFR <30 mL/min/1.73 m^2^ (≥Stage 3b), supporting the FDA’s recommendation to avoid metformin in patients whose eGFR is below this threshold.^13^, ^20^ Moreover, there are increasing reports of LA in patients taking metformin.^21^ The present study sought to extend those findings by evaluating this potential association in patients with established CKD in a real‐world setting and confirm potential risks using different methodologic strategies. To assess this, a nested case‐control study was performed in a population‐based sample of US Veterans to (a) compare risk‐adjusted rates of LA among metformin‐exposed and metformin‐unexposed patients with type 2 diabetes and CKD and (b) assess the association between metformin exposure and risk of LA across the spectrum of degrees of kidney dysfunction.

## 2 METHODS

### 2.1 Study design

This study was a nested case‐control study that used administrative claims and electronic medical record data from the national VA Corporate Data Warehouse (CDW) from 2003 to 2013. The CDW includes all data from Veterans Health Information Systems and Technology Architecture (VistA), inpatient and outpatient administrative data sets (MedSAS), cost information (DSS) and other non‐VistA data through Text Integration Utilities from 1243 healthcare facilities, including 170 Veterans Affairs (VA) Medical Centers and 1063 outpatient sites. Data captured in the CDW include inpatient and outpatient diagnosis/procedure codes, pharmacy, vital sign and laboratory data. This study was approved by the VA North Texas Health Care System and Texas Tech University Health Sciences Center Institutional Review Boards prior to data acquisition.

### 2.2 Study cohort

A sample of eligible patients from a base cohort is necessary to conduct a nested case‐control study. This base cohort was assembled consisting of all adult patients (≥18 years) with type 2 diabetes and CKD treated at VA medical centres with a prescription for any antihyperglycaemic medication during FY 2003‐13. Patients were excluded if they did not have a prescription for an antihyperglycaemic medication. Type 2 diabetes was identified using a validated algorithm that uses both administrative claims and pharmacy data.^22^ Briefly, type 2 diabetes was identified when a patient received a prescription for an antihyperglycaemic medication and/or two or more diabetes diagnostic codes from inpatient and/or outpatient visits over a 24‐month period prior to base cohort entry. Chronic kidney disease status was identified using a validated algorithm that uses a combination of International Classification of Diseases, Ninth Revision (ICD‐9‐CM) codes from all healthcare encounters (diagnosis groups: chronic renal insufficiency, diabetic nephropathy, hypertensive nephropathy, acute renal failure and miscellaneous other renal disease) or clinical laboratory data (eGFR < 60 mL/min/1.73 m^2^ for 2 consecutive readings >3 months apart).^23^ The CKD‐EPI equation was used to calculate eGFR.^24^ The CKD classifications were determined at base cohort entry in accordance with Kidney Disease: Improving Global Outcomes CKD Work Group.^25^ Patients with an eGFR ≥90 mL/min/1.73 m^2^ were classified as Stage 1, 60‐89 mL/min/1.73 m^2^ were classified as Stage 2, 45‐59 mL/min/1.73 m^2^ were classified as Stage 3a, 30‐44 mL/min/1.73 m^2^ were classified as Stage 3b, 15‐29 ml/min/1.73 m^2^ were classified as Stage 4, and <15 mL/min/1.73 m^2^ were classified as Stage 5. Patients were excluded if they received dialysis or had a kidney transplant as identified by inpatient or outpatient ICD‐9‐CM or CPT codes prior to cohort entry. Base cohort entry began when patients met the criteria for both type 2 diabetes and CKD regardless of which disease presented first.

### 2.3 Nested case‐control

Nested case‐control analyses were conducted within the base cohort. This approach was chosen because of the size of the overall cohort and the time‐varying nature of exposure to metformin.^26^ Risk‐set sampling was used for the matching of controls to cases, which produces odds ratios that are unbiased estimators of hazard ratios (HRs).^27^

Cases consisted of all patients with a hospital admission (lasting at least one day) with LA during follow‐up after cohort entry (ICD‐9‐CM 276.2 in either the primary or secondary position of the discharge diagnosis) or inpatient serum lactate >5 mEq/L.^28^, ^29^ For each case, the index date was defined by the date of hospital admission.

Risk‐set sampling was used to randomly select up to 10 controls for each case, matched on age (±365 days), sex, date of entry to the nested case‐control study (±180 days) and duration of diagnosed diabetes before entry to the nested case‐control study (defined as time between entry to the base cohort and entry to the nested case‐control study; ± 90 days). Matched controls were assigned the index date of their respective cases.

### 2.4 Exposure assessment

Cases and controls were classified using two exposure definitions based on pharmacy fills at index date:

1. Metformin exposure within 3 months up to index date or current use.
2. Metformin exposure anytime within 6 months of index date.

Current use was defined as metformin prescription fill plus a 30‐day grace period overlapping the index date. The primary analysis was conducted on metformin exposure within 3 months up to index date and current use due to the proximity to the index event of LA.

### 2.5 Confounder assessment

In addition to age, calendar year of cohort entry, sex, duration of diagnosed diabetes and duration of follow‐up on which the models were conditioned, the following potential baseline characteristics, assessed at the study cohort entry date, were adjusted for diagnoses of atrial fibrillation or flutter, carotid revascularization, prior myocardial infarction, peripheral artery disease, depression, post‐traumatic stress disorder and schizophrenia by ICD‐9‐CM. Moreover, the models were adjusted for the following characteristics assessed at the index date: eGFR as a continuous measure, diagnosis of cancer, heart failure, chronic obstructive pulmonary disease, asthma, human immunodeficiency virus (HIV), hepatic failure, obstructive coronary disease, respiratory failure, stroke, transient ischaemic attack, dementia and sepsis by ICD‐9‐CM codes. These variables were assessed at the index date because literature suggests that they are potential common causes (or proxies of common causes) of both metformin use and LA but not potential consequences of metformin use. Concomitant medication exposure was assessed at the index date and included antihypertensive medications, anti‐arrhythmic medications, anticoagulants, antipsychotic medications, nitrates, statins, isoniazid and HIV medications. Supplemental Table S1 provides the ICD‐9‐CM codes and other algorithms used to define these variables.^30^

### 2.6 Statistical analyses

The crude incidence rate of LA in the full study cohort was calculated with 95% confidence intervals (CIs) based on the Poisson distribution. The incidence of LA in the full cohort was calculated by metformin exposure status at the time of entry into the base cohort. Likewise, in the nested case‐control sample, person‐years were calculated based on exposure status in the previous three months.^26^ Rates were calculated in the nested case‐control using person‐years from the nested case‐control population and corresponding sampling fraction from person‐years of the full cohort.^26^ Rate differences were calculated by multiplying person‐years in the sampling fraction by the rate ratio. The 95% CIs were calculated using the Poisson distribution. Conditional logistic regression was used to estimate hazard ratios (HRs) and corresponding 95% CIs of LA, comparing metformin current and within 3 months use versus current use of other oral antihyperglycaemic medications. If a patient was exposed to metformin in combination with other oral antihyperglycaemic medications, they were considered metformin‐exposed. All models were adjusted for the potential confounders listed above. To account for effect modification by CKD stage, a nested case‐control study was conducted stratifying by CKD stages 1 and 2 combined, 3a, 3b, and 4 and 5 combined.

### 2.7 Propensity score analyses

Cohort analyses matched on propensity score for treatment with metformin were performed to test the robustness of the results and identify potential subgroups of patients at heightened risk for LA when exposed to metformin.^31^ The primary analysis was repeated for patients grouped by CKD stage 1 or 2, CKD stage 3a, CKD stage 3b and CKD stage 4 or 5 at study cohort entry. All patients were censored at 150 days after cohort entry to account for events that occur proximal to the incident exposure. Exposure was defined as being exposed to metformin at base cohort entry. To be categorized as metformin exposed, patients must not have a prescription for metformin in the 1 year prior to base cohort entry and then prescribed metformin at base cohort entry. Unexposed patients must not have a prescription for metformin in the 1 year prior to base cohort entry and then not prescribed metformin, rather prescribed another antihyperglycaemic medication at base cohort entry. The intent‐to‐treat principle was utilized to categorize exposure during follow‐up. Logistic regression models were used to create the propensity score for metformin exposure, which modelled the probability of metformin use given 36 study covariates at baseline.^32^ These candidate covariates were selected based on previous literature.^19^, ^30^ Nearest‐number matching was performed with a calliper of 0.0001. Patients treat with metformin were matched 1:1 to patients receiving other antihyperglycaemic medications at base cohort entry. Standardized differences were calculated to assess the balance of covariates in the propensity score‐matched groups. The standardized difference compares the difference in means in units of the pooled standard deviation.^33^ Unlike tests of statistical hypothesis, the standardized difference is not influenced by sample size. Propensity‐matched cohort subanalyses were conducted for patients grouped with CKD stages 1 or 2, CKD stage 3a, CKD stage 3b and CKD stages 4 or 5. Standardized differences were calculated to evaluate any differences between groups. Metformin dose was assessed in the metformin‐exposed sample within the propensity‐matched cohort. Metformin daily dose was stratified to less than 500mg (reference), 501‐1,000mg, 1,001‐1,500mg, 1,501‐2000mg, greater than 2,000mg. Statistical analyses were conducted using SAS, version 9.4 (SAS Institute).

### 2.8 Role of the funding source

The funding sources had no role in the study's design, conduct and reporting.

## 3 RESULTS

**FIGURE 1:**
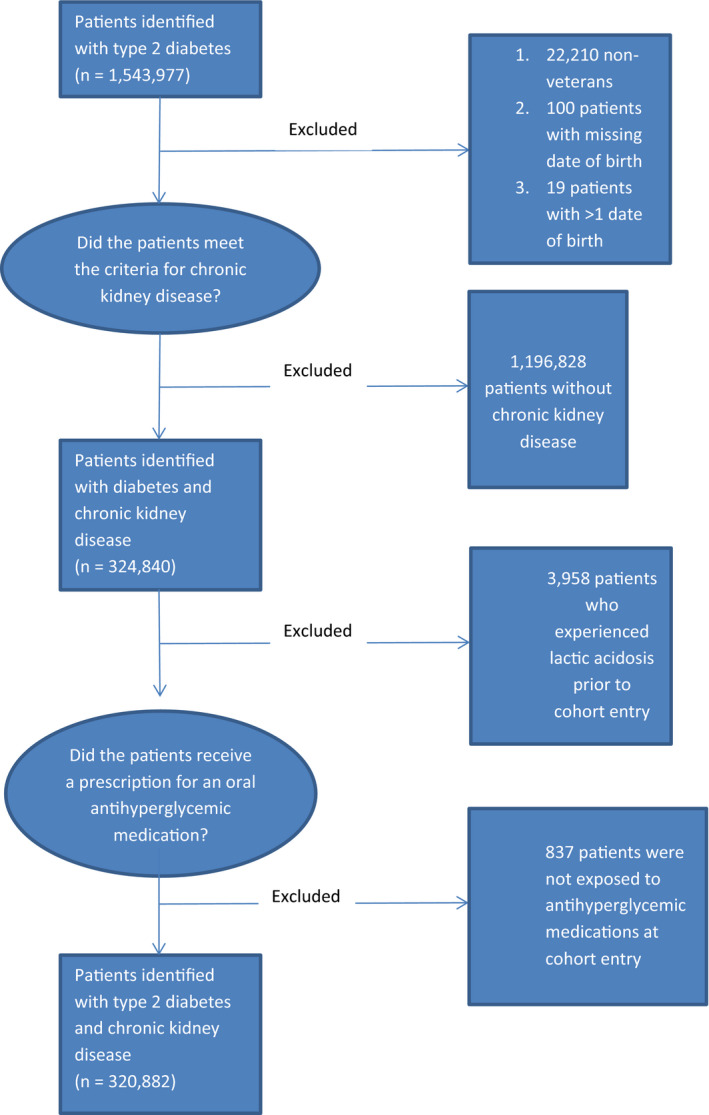
Construction of the Base Cohort

A total of 320 882 patients met criteria for inclusion into the base cohort (Figure 1). The average age at cohort entry was 70.5 years (Standard deviation [SD] 10.3), and 313,271 were male (97.6%). The overall cohort had a mean follow‐up of 4.2 years for a total of 1,331,784 patient‐years of observation. During this time, 2,665 patients experienced LA generating a crude incidence rate of 2.00 (95% CI 1.93‐2.08) per 1000 person‐years. The crude LA rate in the base cohort was 1.81 (95% CI 1.67‐1.96) per 1,000 person‐years in the metformin‐exposed group and 2.07 (95% CI 1.98‐2.16) per 1000 person‐years in the metformin‐unexposed group.

### 3.1 Nested case‐control study

Table 1 presents the baseline and event‐time characteristics of the 2,662 cases and 26,602 matched controls. The cases had more peripheral artery disease (23.7% vs 17.3%), cancer (29.1% vs 19.5%), heart failure (20.7% vs 8.8%), liver failure 10.9% vs 2.7%), respiratory failure (18.7% vs 5.4%), stroke (11.5% vs 5.5%) and sepsis (2.6% vs 0.02%). Cases also had a higher prevalence of exposure to HIV medications (0.9% vs 0.5%) and isoniazid (0.2% vs 0.1%), both of which are associated with LA. Table 2 describes the incidence rates for those included in the nested case‐control study.

**TABLE 1:** Baseline and event‐time characteristics for matched cases and controls

Characteristic	Cases (2,662) n (%)	Controls (26,602) n (%)
Age, mean (SD)	67.4 ± 10.8	67.7 ± 10.3
Sex		
Male	2584 (97.1)	25 880 (97.3)
Race		
White	1793 (67.4)	19 027 (71.5)
Black	625 (23.5)	3940 (14.8)
Asian	11 (0.4)	152 (0.6)
Other	107 (4.0)	1024 (3.9)
Unknown	126 (4.7)	2459 (9.2)
Baseline Characteristics		
Atrial fibrillation	358 (13.5)	3029 (11.4)
Other arrhythmia	143 (5.4)	1064 (4.0)
Prior carotid revascularization	76 (2.9)	465 (1.8)
Prior myocardial infarction	169 (6.4)	1355 (5.1)
Peripheral artery disease	631 (23.7)	4590 (17.3)
Depression	639 (24.0)	5623 (21.1)
Post‐traumatic stress disorder	286 (10.7)	2435 (9.2)
Schizophrenia	70 (2.6)	488 (1.8)
Event‐time Characteristics		
Cancer	775 (29.1)	5198 (19.5)
Heart failure	550 (20.7)	2346 (8.8)
COPD/asthma	712 (26.8)	5379 (20.2)
HIV	26 (1.0)	151 (0.6)
Liver Failure	291 (10.9)	721 (2.7)
Obstructive coronary disease	1396 (52.4)	12 078 (45.4)
Respiratory failure	497 (18.7)	1445 (5.4)
Stroke	306 (11.5)	1451 (5.5)
Transient ischaemic attack	85 (3.2)	410 (1.5)
Dementia	180 (6.8)	1293 (4.9)
Sepsis	69 (2.6)	6 (0.02)
CKD Staging		
1	52 (2)	524 (2)
2	341 (12.8)	6,304 (23.7)
3	1,077 (40.5)	15,816 (59.5)
4	644 (24.9)	2,528 (9.5)
5	528 (19.8)	1,430 (5.4)
By Year		
2002	17 (0.6)	405 (1.5)
2003	152 (5.7)	1965 (7.4)
2004	203 (7.6)	2353 (8.9)
2005	275 (10.3)	2765 (10.4)
2006	312 (11.7)	3263 (12.3)
2007	327 (12.3)	3214 (12.1)
2008	291 (10.9)	2998 (11.3)
2009	306 (11.5)	3091 (11.6)
2010	300 (11.3)	2803 (10.5)
2011	237 (8.9)	2189 (8.2)
2012	182 (6.8)	1324 (5.0)
2013	60 (2.3)	232 (0.9)
Event‐time Medication Exposure		
Anti‐arrhythmic medications	338 (12.7)	2188 (8.2)
Anticoagulant medications	494 (18.6)	2753 (10.4)
Antipsychotic medications	132 (5.0)	775 (2.9)
Antihypertensive medications		
Beta‐blockers	1775 (66.7)	12 870 (48.4)
Calcium channel blockers	1172 (44.0)	8717 (32.8)
Thiazide diuretics	1007 (37.8)	8367 (31.5)
Loop diuretics	1267 (47.6)	6791 (25.5)
ACE inhibitors	1470 (55.2)	12 267 (46.1)
Nitrates	383 (14.4)	2133 (8.0)
Other	746 (28.0)	5693 (21.4)
Statin medications	1725 (64.8)	15 391 (57.9)
Isoniazid	4 (0.2)	35 (0.1)
HIV medications	24 (0.9)	119 (0.5)

Abbreviations: ACE, angiotensin‐converting enzyme; CKD, chronic kidney disease; COPD, chronic obstructive pulmonary disorder; HIV, human immunodeficiency virus; SD standard deviation.

**TABLE 2:** Nested case‐control analysis (N = 29,264; n (cases) = 2,662, n(controls) = 26,602)

Metformin exposed	Metformin exposed, n	Lactic acidosis (LA) event	Person‐years (PY)*	Incidence rate of LA per 1,000 PY**	95% CI	Incidence rate ratio	Rate Difference per 1,000 person‐years
Yes	2791	227	5665	2.01	1.75‐2.28	1.57	0.04
No	26 473	2385	60 984	1.96	1.88‐2.04	Reference	Reference

* Person‐years calculated based on exposure status in the previous three months.

** Rates calculated using person‐years from nested case‐control and corresponding sampling fraction from 1 331 783 person‐years of the full cohort.

In the nested case‐control analysis, there were 3 cases for whom a matched control could not be identified; excluding these, the estimated LA rate was 2.00 (95% CI 1.92‐2.08) per 1,000 patient‐years. The estimated event rate for patients exposed to metformin was 2.01 (95% CI 1.75‐2.28) per 1,000 person‐years and 1.96 (96% CI 1.88‐2.04) per 1,000 person‐years for matched patients not exposed to metformin (Table 3).

**TABLE 3:** Adjusted hazards ratios of metformin association with lactic acidosis in a nested case‐control study stratified by CKD stage

Variable	Adjusted Hazard Ratio*	95% CI
Current Metformin exposure at event/matched control		
CKD stage 1 or 2	0.83	0.54‐1.27
CKD stage 3a	2.26	1.59‐3.19
CKD stage 3b	3.69	2.11‐6.43
CKD stage 4 or 5	5.96	2.62‐13.57
Metformin exposure within 3 months prior to event/matched control		
CKD stage 1 or 2	1.05	0.71‐1.57
CKD stage 3a	3.09	2.19‐4.35
CKD stage 3b	3.34	1.95‐5.72
CKD stage 4 or 5	7.87	3.51‐17.61
Metformin exposure within 6 months prior to event/matched control		
CKD stage 1 or 2	1.04	0.72‐1.52
CKD stage 3a	2.24	1.58‐3.16
CKD stage 3b	3.85	2.34‐6.36
CKD stage 4 or 5	5.19	2.66‐10.13

* Adjusted for baseline characteristics: diagnoses of atrial fibrillation, arrhythmia, carotid revascularization, myocardial infarction, peripheral artery disease, depression, post‐traumatic stress disorder, and schizophrenia. Also adjusted for event time characteristics: eGFR, diagnosis of cancer, heart failure, chronic obstructive pulmonary disease, asthma, human immunodeficiency virus infection, liver failure, obstructive coronary disease, respiratory failure, stroke, transient ischaemic attack, dementia, and sepsis. Medications adjusted for at event time: antihypertensive medications, anti‐arrhythmic medications, anticoagulants, antipsychotic medications, nitrates, statins, isoniazid, and HIV medications.

When compared with other antihyperglycaemic medications, metformin exposure was associated with a higher hazard of LA if exposed within the previous 3 months (HR 1.97, 95% CI 1.69‐2.29) or previous 6 months (HR 1.86, 95% CI 1.60‐2.15) for all CKD groups combined. In patients with CKD stage 1 or 2, metformin exposure was not significantly associated with the hazard of LA if they were currently exposed (HR 0.83, 95% CI 0.54‐1.27), exposed in the previous 3 months (HR 1.05, 95% CI 0.71‐1.57) or previous 6 months (HR 1.04, 95% CI 0.72‐1.52). For patients with CKD stage 3a, there was a higher rate of LA in those currently exposed to metformin (HR 2.26, 95% CI 1.59‐3.19), in the prior 3 months (HR 3.09, 95% CI 2.19‐4.35) or 6 months (HR 2.24, 95% CI 1.58‐3.16). In patients with CKD stage 3b, there was a higher rate of LA in those currently exposed to metformin (HR 3.69, 95% CI 2.11‐6.43), in the prior 3 months (HR 3.34, 95% CI 1.95‐5.72) or 6 months (HR 3.85, 95% CI 2.34‐6.36). In the subgroup of patients with CKD stage 4 and 5, metformin exposure had the strongest association with LA if currently exposed (HR 5.96, 95% CI 2.62‐13.57), exposed within the prior 3 months (HR 7.87, 95% CI 3.51‐17.61) or 6 months (HR 5.19, 95% CI 2.66‐10.13) (Table 3).

### 3.2 Propensity score‐matched cohort study

A total of 73,510 patients exposed to metformin were matched to 73,510 patients not exposed to metformin (Supplemental Table S2). In both groups, the majority of patients were white (76.2% vs 75.7%, respectively) and male (97.4% vs 97.7%, respectively). The mean age in the matched cohort was 67.8 (SD 9) years in the metformin‐exposed group and 67.9 (SD 9.7) years in the unexposed group. Supplemental Table S2 shows the cohort to be well balanced between groups after propensity score matching. Metformin was associated with LA (RR 1.38, 95% CI 1.08‐1.77) when compared with unexposed patients across all CKD groups (Supplemental Table S3). There was no higher risk of LA in patients with versus without metformin exposure with CKD stage 1 and 2 (RR 0.97, 95% CI 0.24‐3.90) or CKD stage 3a (RR 1.05, 95% CI 0.76‐1.44). Patients exposed to metformin had a higher risk of LA with CKD stage 3b (RR 1.86, 95% CI 1.06‐3.25) and CKD stage 4 and 5 (RR 2.34, 95% CI 1.28‐4.26). When restricted to only those exposed to metformin, total daily dose did not have an impact on the risk of LA (Supplemental Table S4).

## 4 DISCUSSION

In this large, observational study of US veterans treated in the VA Health System with T2DM and CKD, the overall absolute rate of LA was 2 events per 1000 patient‐years, higher in the metformin‐exposed compared with unexposed patients. Metformin was not associated with the development of LA in those with CKD stages 1‐2. Rather, the association between metformin use and LA was present only in those with CKD stages 3a and higher, and the association got stronger as the CKD stage increased. This is the largest observational study examining the risk of LA in patients with T2DM and CKD, which allows us to obtain some of the most precise estimates of association to date for this population.

The absolute LA incidence rate in this study is similar to that seen in prior large systematic reviews.^14^, ^15^, ^17^ Moreover, these results are consistent with the findings of a large cohort study where metformin was significantly associated with LA in those with eGFR < 60 mL/min/1.73 m^2^ (7.4 per 100,000 person‐years in metformin users vs. 2.2 per 100,000 person‐years in nonusers; adjusted HR 6.37, 95% CI 1.48‐27.5).^34^ These results are discordant with the results of large cohort studies evaluating data from the Geisinger Health System and from MarketScan.^19^ Lazarus et al found that time‐dependent metformin use was not associated with incident acidosis overall (adjusted HR, 0.98; 95% CI, 0.89‐1.08) or in patients with eGFR 45 to 59 mL/min/1.73 m^2^ (adjusted HR 1.16, 95% CI 0.95‐1.41) or eGFR 30 to 44 mL/min/1.73 m^2^ (adjusted HR 1.09, 95% CI 0.83‐1.44). However, they did find metformin use to be associated with a higher risk of acidosis at eGFR < 30 mL/min/1.73 m^2^ (adjusted HR, 2.07; 95% CI 1.33‐3.22). This is consistent with our findings of a higher risk of LA in patients with stage 4 or 5 CKD, although our analyses revealed a greater magnitude association in this group than did Lazarus et al This may be explained by the inherent differences between patients in a VA population and those in the private sector or by some inherent imprecision of risk estimates due to the underlying small numbers of actual events in some studies. Veterans are, for the most part, an older and sicker cohort with more multimorbidity compared with the general population, which may also explain some of the discordance in published results. However, despite the differences in the magnitude of the associations, this study also found that the relative risk for metformin associated LA was higher in those with more advanced kidney disease. Lalau et al conducted a metformin dose finding study in 78 patients with CKD stages 3A/3B and 4.^35^ These investigators found that the appropriate daily dosing schedules were 1500 mg in CKD stage 3A, 1000 mg in CKD stage 3B and 500mg in CKD stage 4. These recommendations, while reasonable, were based on metformin concentrations and not LA risk. Moreover, the investigators found that elevated lactate values were not chronologically related to metformin concentrations.

This study is in contrast to the study by Ekstrom et al^36^ In their study of 51,675 Swedish patients with T2DM, they found that metformin, when compared to other diabetes treatments, reduced acidosis/serious infection in patients with an eGFR 45 to 60 ml/min/1.73 m^2^ (HR 0.85, 95% CI 0.74 to 0.97) and had no increased risk in patients with an eGFR 30 to 45 ml/min/1.73 m^2^ (HR 0.98, 95% CI 0.79 to 1.21). This result may be confounded since 19 variables were included in the propensity score model, and patients that were on metformin monotherapy was the exposure of interest. This may introduce a healthy user bias since patients on metformin monotherapy are often have less diabetes complications and burden of illness.^37^

These findings support the FDA’s expansion of the use of metformin in patients with eGFR as low as 30 mL/min/1.73 m^2^ (Stage 3b), the threshold for contraindication on the current US product labels for metformin and its fixed dose combinations. Although these results demonstrated a higher risk of LA in patients with stage 3a or 3b CKD, the differences in the absolute rates of LA in those with versus without metformin exposure were quite modest. Nonetheless, caution is warranted and these observations are in harmony with the FDA product labelling encouraging metformin dose reduction and discourage its initiation as patients progress into CKD stage 3b. This is in contrast to the findings in patients with stage 4 or 5, where the LA risk was multiple times higher in patients exposed to metformin, where metformin remains appropriately contraindicated.

Metformin is recommended by most professional society treatment algorithms as the first line therapy in patients with T2DM absent contraindications or intolerance due to its wide availability, neutral effect on weight, low risk of hypoglycaemia and affordability when compared with other antihyperglycaemic medications.^1^, ^3^, ^6^, ^38^ Therefore, any demonstrated risks associated with metformin in patients with CKD should be considered in the overall context of efficacy and impact of glycaemic control.

Several limitations of the present study are noteworthy. As in any observational study, there is potential for residual or unmeasured confounding and biases unaccounted for. To address this concern, multiple potential confounders were adjusted for and a propensity score‐matched analyses were conducted to assess the robustness of the results. Moreover, a nested case‐control study was conducted to reduce the risk of immortal time bias and account for the time‐varying nature of metformin exposure. This study did not assess arterial pH in our definition of LA. This may misclassify the outcome; however, using arterial pH in the definition of LA in observational studies has not been validated and may further misclassify events. The diagnosis code and lactate levels have been used in other observational studies and may distinguish if metformin contributed to LA from other metabolic factors.^19^, ^36^, ^39^ The US veteran population is overwhelmingly white men; therefore, the generalizability of the observations to more heterogeneous populations is uncertain. However, the nested case‐control study had almost 1000 women and almost 9000 nonwhite patients. Also, this study did not assess the effect of acute kidney injury (AKI) on the risk of metformin induced LA in the setting of CKD. Therefore, caution should be exercised when dosing metformin in this setting since there is limited evidence. Similarly, there was an increased use of loop diuretics in the LA cases compared to the controls. This may cause AKI through dehydration and confound the effect of metformin on LA. Loop diuretics were adjusted for in both the multivariate and propensity score models, and given the similar results in both models, there is a lower likelihood that this confounding had a major impact. Ascertainment bias may be present in patients with CKD stage 3 and greater. Clinicians may draw a lactic acid plasma concentration in patients with higher degrees of renal insufficiency and an active metformin prescription. However, we know of no plausible mechanism by which such diagnostic suspicion bias could be responsible for the studies primary finding that there was no association between metformin and LA in persons with CKD stage 1 or 2. There was a discrepancy in findings between the nested case‐control study and propensity‐matched cohort study with regard to those patients with CKD stage 3a. This may be a product of the intent‐to‐treat principle where patients who were initiated on metformin at study cohort entry may have stopped or switched therapy in the follow‐up period, thereby decreasing LA risk in patients in the propensity‐matched cohort study.

In conclusion, metformin was not associated with an elevated risk of LA in persons with stage 1‐2 CKD, but was associated with higher risks of LA at more advanced stages of CKD. The highest risk was in patients with stage 4 or 5 CKD, and thus, this agent is appropriately contraindicated in this group. This absolute difference in risk was very small in patients with mild or moderate CKD but higher in CKD stage 3b than 3a; therefore, cautious use and consideration of dose reduction in patients progressing from stage 3a to 3b is appropriately recommended by contemporary US product labelling.

## CONFLICTS OF INTEREST

All investigators report no conflicts of interest.

## AUTHOR CONTRIBUTIONS

CAA, EAH, MJP, DKM, SH, RTM, IL and EM conceived the concept for the study. CAA, EAH, MJP, DKM, SH, IL and EM wrote the manuscript. HY and MC extracted the data from VINCI. CAA, SMV, AZ, HY and MC analysed the data. All authors contributed to the discussion and reviewed/edited the manuscript.

## Supporting information

Table S1‐S4

## Data Availability

All data are stored on VA servers and archived. These data belong to the veterans and therefore cannot share these data per VA policies.
