# Supplementary material for: Lactic acidosis incidence with metformin in patients with type 2 diabetes and chronic kidney disease: A retrospective nested case‐control study
Source: Endocrinol Diabetes Metab. 2020 Jul 17;4(1):e00170. doi: 10.1002/edm2.170 (PMC7831229; doi:10.1002/edm2.170)
Supplement: Supplementary file 1 — Table S1‐S4 [file EDM2-4-e00170-s001.docx]

**Table S1.**

| **Covariate** | **Conditions** | **Definition** |
| --- | --- | --- |
| **Malignancy** | Cancer excluding non  melanoma skin cancer | ICD 9- CM diagnosis codes:140.X-208.X (exclude 173) |
| **Liver and Respiratory failure** | End Stage Liver disease  Respiratory failure | ICD 9- CM diagnosis codes: 570.X- 573.X  ICD 9- CM diagnosis codes: 518.81, 518.83, 518.84, 799.1, 415.X, 416.X |
| **Heart Failure** | HF excluding post procedure-HF | ICD 9- CM diagnosis codes: 428.X, 402.01, 402.11, 402.91, 404.01,  404.03, 404.11, 404.13, 404.91, 404.93, 425.X |
| **Cardiovascular Disease** | 1. MI 2. Obstructive coronary disease 3. TIA 4. Stroke 5. Peripheral artery disease, revascularization or amputation 6. Carotid revascularization 7. Pentoxifylline and related medications | ICD 9- CM diagnosis codes:410.X, 412.X, 429.7X  ICD 9- CM diagnosis codes:411.X, 413.X, 414.X  ICD9-CM procedure codes: 36.01, 36.02, 36.03, 36.05, 36.09, 36.10-  36.19  CPT procedure codes: 33533-36, 33510-23, 33530, 92980-82,92984,  92995-6, 92974  ICD 9- CM diagnosis codes: 435.X  ICD 9- CM diagnosis codes: 430.X, 431.X. 434.X, 436.X  ICD 9- CM diagnosis codes:440.2X, 442.2, 443.1, 443.9, 445.0X ICD9-CM  procedure codes:38.08-09, 38.18, 38.38, 38.39, 38.48, 38.49, 38.88, 38.89, 39.25, 39.29, 39.5, 84.1X; 84.10-84.17  CPT procedure codes: 35226,35256, 35286, 35351, 35355, 35371, 35372,  35381, 35454, 35456, 35459, 35473, 35474, 35482, 35483, 35485, 35492,  35493, 35495, 35546, 35548, 35549, 35551, 35556, 35558, 35563, 35565,  35566, 35571, 35583, 35585, 35587, 35646, 35651, 35654, 35656, 35661,  35663, 35665, 35666, 35671, 34800, 34802-5  ICD9-CM procedure codes: 38.12, 38.11, 00.61, 00.63, 39.28  CPT procedure codes: 35301, 0005T, 0006T, 0007T, 0075T, 0076T,  37215, 37216  HCPCS procedure code: S2211  Medications: Pentoxifylline, Cilostazol, Cyclandelate, Ethaverine HCL,  Nicotinyl Alcohol Tartate, Papaverine, Tolazolin |
| **Serious Mental illness** | 1. Dementia 2. Depression 3. Schizophrenia 4. Bipolar disorder 5. Post traumatic disorder | ICD 9- CM diagnosis codes: 290.X, 291.2, 292.82, 294.1X, 331.0-  331.1X, 331.82  Medications: Donepezil, Rivastigmine, Galantamine, Tacrine,  Memantine  ICD 9- CM diagnosis codes: 311, 300.4, 296.2, 296.3, V79.0  ICD 9- CM diagnosis codes: 295.X  ICD 9- CM diagnosis codes: 296.0, 296.4X, 296.5X, 296.6X, 296.7,  296.80, 296.89  ICD 9- CM diagnosis codes: 309.81 |
| **Cardiac valve disease** |  | ICD 9- CM diagnosis codes: 394.X, 395.X, 396.X, 424.0, 424.1 |
| **Arrhythmia** |  | ICD 9- CM diagnosis codes: 427.3X  ICD 9- CM diagnosis codes: 426.X, 427.X |
| **Smoking** |  | ICD 9- CM diagnosis codes:305.1, V15.82, 989.84  Medications: Varenicline tartrate, Nicotine Replacement therapy  (gum, patch, lozenge) |
| **COPD/Asthma** |  | ICD 9- CM diagnosis codes:491.X, 492.X, 493.X, 496.X, V17.5,  V81.3 |
| **HIV** |  | ICD 9- CM diagnosis codes: 042, 079.53, 795.71, V08  Medications: Zidovudine, Didanosine, Zalcitabine, Stavudine,  Indinavir, Ritonavir, Saquinavir, Nevirapine, Nelfinavir, Delavirdine,  Delavirdine, Abacavir, Amprenavir, Efavirenz, Lamivudine-  Zidovudine, Ritonavir-Lopinavir, Abacavir-Lamivudine-Zidovudine |
| **Parkinson’s Disease** |  | ICD 9- CM diagnosis codes: 332  Medications: Apokyn, Apomorphine, Carbidopa/levodopa,  Entacapone, Pergolide, Pramipexole, Ropinirole, Rotigotine,  selegiline, Tolcapone, Zelapar Azilect/rasagiline, Emsam,  Isocarboxazid, Phenelzine, Tranylcypromine |
| **ACE Inhibitors or ARBs** | alone or in combination | Benazapril, Captopril, Enalapril, Fosinopril, Lisinopril, Moexipril,  Perindopril, Quinapril, Ramipril, Trandolapril, Candesartan, Eprosartan,  Irbesartan, Losartan, Azilsartan, Olmesartan, Telmisartan, Valsartan |
| **Beta blockers** | alone or in combination | Acebutolol, Atenolol, Betaxolol, Bisoprolol, Carteolol, Carvedilol,  Esmolol, Labetalol, Metoprolol Tartrate, Metoprolol Succinate, |
| **Calcium Channel Blockers** | alone or in combination | Amlodipine, Isradipine; Felodipine, Nifedipine, Nifedipine ER,  Nicardipine; Diltiazem, Verapamil, Nimodipine; Nisoldipine; Bepridil,  Amlodipine–Atorvastatin, Clevidipine Butyrate, |
| **Thiazide/ potassium sparing diuretics** | alone or in combination | Chlorothiazide, Chlorthalidone, Hydrochlorothiazide, Methyclothiazide,  Trichlormethiazide, Metolazone, Indapamide, Eplerenone; Ameloride,  Sprinolactone, Triamterene, Hydrochlorothiazide/Triamterene,  Hydrochlorothiazide/Spironolactone, Bendroflumethiazide, Benzthiazide,  Cyclothiazide, Hydroflumethiazide, Methyclothiazide, Trichlormethiazide,  Metolazone, Indapamide, Polythiazide, Quinethazone |
| **Other Antihypertensives** | alone or in combination | Doxazosin, Prazosin, Terazosin, Clonidine, Guanabenz, Guanfacine,  Hydralazine, Methyldopa, Metyrosine, Reserpine, Minoxidil, Alfuzosin,  Silodosin, Alseroxylon, Cryptenamine, Deserpidine, Diazoxide  Guanethidine, Iloprost, Mecamylamine, Pargyline, Rescinnamine,  Trimethaphan Camsylate |
| **Digoxin** |  | Digoxin, Digitalis |
| **Antiarrhythmics** |  | Adenosine, Amiodarone, Lidocaine, Flecainide, Ibutilide, Pacerone,  Procainamide, Rhythmol, Propafenone, Quinidine, Disopyramide,  Verapamil, Dofetilide, Mexiletine, Moricizine, Tocainide |
| **Anticoagulants** |  | Warfarin, Argatroban, Bivalirudin, Dalteparin, Enoxaprin, Eptifibatide,  Fondaparinux, Heparin, Lepirudin, Tirofiban, Tinzaparin, Reviparin,  Nadroparin, Ardeparin, Certoparin, Dabigatran |
| **Platelet Inhibitors** |  | Clopidogrel, Ticlopidine, Aspirin/ Dipyrimidole, Dipyrimidole alone,  Abciximab, Factor IX, Factor VIIa, Factor VIII, Prasugrel, Ticagrelor |
| **Statins** |  | Atorvastatin, Fluvastatin, Lovastatin, Pravastatin, Simvastatin,  Rosuvastatin, Cerivastatin Pitavastatin, Lovastatin ER,  Ezetamibe/Simvastatin, Lovastatin /Niacin |
| **Non-statin cholesterol medications** |  | Cholestyramine, Colesevelam, Clofibrate, Colestipol, Niacin,  Niacinamide, Fish Oil Concentrate, Omega 3 Fatty Acids, Gemfibrozil,  Fenofibrate, Fenofibric Acid, Ezetimibe Omacor, Tricor/Fenofibrate, |
| **Nitrates** |  | Amyl nitrate, Isosorbide Dinitrate, Isosorbide mononitrate, Erythrityl  Tetranitrate Nitroglycerin (all forms--SA, Patch, SL, Ointment; Aerosol  spray), Ranolazine |
| **Aspirin** |  | Aspirin, Aspirin/ Dipyrimidole |
| **Loop diuretics** |  | Furosemide, Ethacrynic acid, Bumetanide, Torsemide |

ICD 9- CM= International Classification of Diseases, Ninth Revision, CHF=congestive heart failure, MI=myocardial infarction, TIA=transient ischemic attack, COPD=chronic obstructive pulmonary disease, HIV=human immunodeficiency virus, ACE=angiotensin converting enzyme, ARB=angiotensin receptor blocker

Table S2: Propensity Matched Cohort

| **Characteristic**  **N (%)** | **Metformin Exposed**  **(73,510)** | **Metformin Non-Exposed**  **(73,510)** | **Standardized Difference** |
| --- | --- | --- | --- |
| Age mean (+/- SD) | 67.9 +/- 9.0 | 67.9 +/- 9.7 | 0.001 |
| Diabetes Duration (Days, Mean +/- SD) | 1048 +/- 899.0 | 1055 +/- 1062 | 0.007 |
| Sex  Male | 71580 ( 97.4 ) | 71804 ( 97.7 ) | 0.020 |
| Race  White  Black  Asian  Other  Unknown | 56034 (76.2)  7344 (10.0)  383 (0.5)  2709 (3.7)  7040 ( 9.6 ) | 55666 (75.7)  7664 (10.4)  348 (0.5)  2700 (3.7)  7132 ( 9.7 ) | 0.012  0.014  0.007  0.001  0.004 |
| Baseline Characteristics |  |  |  |
| Cancer | 9512 ( 12.9 ) | 9571 ( 13.0 ) | 0.002 |
| Liver Failure | 1741 ( 2.4 ) | 1734 ( 2.4 ) | 0.001 |
| Respiratory failure | 1793 ( 2.4 ) | 1851 ( 2.5 ) | 0.005 |
| Heart failure | 3169 ( 4.3 ) | 3286 ( 4.5 ) | 0.008 |
| Prior myocardial infarction | 3308 ( 4.5 ) | 3343 ( 4.6 ) | 0.002 |
| Obstructive coronary disease | 26817 ( 36.5 ) | 27250 ( 37.1 ) | 0.012 |
| Transient ischemic attack | 1237 ( 1.7 ) | 1267 ( 1.7 ) | 0.003 |
| Stroke | 3598 ( 4.9 ) | 3654 ( 5.0 ) | 0.004 |
| Peripheral artery disease | 13098 ( 17.8 ) | 13362 ( 18.2 ) | 0.009 |
| Prioro carotid revascularization | 1482 ( 2.0 ) | 1466 ( 2.0 ) | 0.002 |
| Dementia | 1850 ( 2.5 ) | 1908 ( 2.6 ) | 0.005 |
| Depression | 15711 ( 21.4 ) | 15662 ( 21.3 ) | 0.002 |
| Schizophrenia | 919 ( 1.3 ) | 940 ( 1.3 ) | 0.003 |
| Bipolar Disorder | 1415 ( 1.9 ) | 1428 ( 1.9 ) | 0.001 |
| PTSD | 7459 ( 10.2 ) | 7444 ( 10.1 ) | 0.001 |
| Atrial Fibrillation | 6975 ( 9.5 ) | 7156 ( 9.7 ) | 0.008 |
| Arrhythmia | 2149 ( 2.9 ) | 2146 ( 2.9 ) | 0.000 |
| Smoker | 12172 ( 16.6 ) | 12300 ( 16.7 ) | 0.005 |
| COPD/asthma | 11521 ( 15.7 ) | 11665 ( 15.9 ) | 0.005 |
| HIV | 214 ( 0.3 ) | 210 ( 0.3 ) | 0.001 |
| Parkinson’s Disease | 986 ( 1.3 ) | 1025 ( 1.4 ) | 0.005 |
| Sepsis | 116 ( 0.2 ) | 113 ( 0.2 ) | 0.001 |
| CKD Stage |  |  |  |
| Stage 1 and 2 | 1833 ( 2.5 ) | 1784 ( 2.4 ) | 0.004 |
| Stage 3A | 53838 ( 73.2 ) | 54016 ( 73.5 ) | 0.005 |
| Stage 3B | 15013 ( 20.4 ) | 15124 ( 20.6 ) | 0.004 |
| Stage 4 and 5 | 2826 ( 3.8 ) | 2586 ( 3.5 ) | 0.017 |
| Baseline Medication Exposure |  |  |  |
| ACE inhibitors | 54367 ( 74.0 ) | 54577 ( 74.2 ) | 0.007 |
| ARB | 10174 ( 13.8 ) | 10295 ( 14.0 ) | 0.005 |
| Antipsychotic medications | 2013 ( 2.7 ) | 2058 ( 2.8 ) | 0.004 |
| Anti-arrhythmic medications | 7084 ( 9.6 ) | 7389 ( 10.1 ) | 0.014 |
| Anticoagulant medications | 7258 ( 9.9 ) | 7492 ( 10.2 ) | 0.011 |
| Antihypertensive medications | 15568 ( 21.2 ) | 15708 ( 21.4 ) | 0.005 |
| Beta-blockers | 40220 ( 54.7 ) | 40427 ( 55.0 ) | 0.006 |
| Calcium channel blockers | 24713 ( 33.6 ) | 24775 ( 33.7 ) | 0.002 |
| Thiazide diuretics | 38089 ( 51.8 ) | 37771 ( 51.4 ) | 0.009 |
| Loop diuretics | 17498 ( 23.8 ) | 17833 ( 24.3 ) | 0.011 |
| Nitrates | 5506 ( 7.5 ) | 5665 ( 7.7 ) | 0.008 |
| Statin medications | 56910 ( 77.4 ) | 56860 ( 77.4 ) | 0.002 |
| Non-Statin medications | 16910 ( 23.0 ) | 16475 ( 22.4 ) | 0.014 |
| Baseline Antihyperglycemic Exposure |  |  |  |
| Sulfonylureas | --- | 37994 ( 51.6 ) | --- |
| Acarbose/miglitol | --- | 651 ( 0.9 ) | --- |
| Thiazolidinediones | --- | 3857 ( 5.3 ) | --- |
| Insulin | --- | 18863 ( 25.7 ) | --- |
| Other* | --- | 12145 ( 16.5 ) | --- |

SD=standard deviation, COPD=chronic obstructive pulmonary disorder, HIV=human immunodeficiency virus, CKD=chronic kidney disease, ACE=angiotensin converting enzyme. *= Dipeptidyl peptidase-4 inhibitors, glucagon-like peptide-1, meglitinides, and sodium-glucose cotransporter 2 inhibitors

Table S3: Risk of Lactic Acidosis in Patients Exposed to Metformin Relative to Other Antihyperglycemic Medications in Propensity Matched Cohorts Restricted to 150 Days After Cohort Entry

| **Group** | **Metformin Exposed**  **N (%)** | **Metformin Non-exposed**  **N (%)** | **Relative Risk** | **95% Confidence Interval** |
| --- | --- | --- | --- | --- |
| Total PS Matched Cohort | 152 (0.21) | 110 (0.15) | 1.38 | 1.08 – 1.77 |
| CKD Stages 1 and 2 | 4 (0.22 ) | 4 (0.22) | 0.97 | 0.24 –  3.90 |
| CKD Stage 3a | 75 (0.14) | 72 (0.13) | 1.05 | 0.76 – 1.44 |
| CKD Stage 3b | 35 (0.23) | 19 (0.13) | 1.86 | 1.06 – 3.25 |
| CKD Stage 4 and 5 | 38 (1.34) | 15 (0.58) | 2.34 | 1.28 – 4.26 |

PS=propensity score, CKD=chronic kidney disease

**Table S4. Risk of Lactic Acidosis in Patients Exposed to Metformin By Daily Dosage Restricted to 150 Days After Cohort Entry (n=75,687)**

| **Metformin Daily Dose** | **Relative Risk** | **95% Confidence Interval** | **P value** |
| --- | --- | --- | --- |
| <500mg | Reference | - | - |
| 501-1,000mg | 0.95 | 0.75-1.20 | 0.64 |
| 1,001-1,500mg | 1.56 | 0.92-2.66 | 0.10 |
| 1,501-2,000mg | 1.13 | 0.89-1.43 | 0.33 |
| >2,000mg | 1.22 | 0.75-1.97 | 0.43 |
